# Efficacy of exercise training for improving vascular dysfunction in people with cancer: a systematic review with meta-analyses

**DOI:** 10.1007/s11764-023-01372-7

**Published:** 2023-04-20

**Authors:** Natalie K. Vear, Yubin Moon, Gregore I. Mielke, Tina L. Skinner, Jeff S. Coombes, Alexandra L. McCarthy, Claudia R. Abbott, Tom G. Bailey

**Affiliations:** 1 School of Nursing, Midwifery and Social Work, The University of Queensland, Level 3, Chamberlain Building (35), St Lucia, Queensland 4072 Australia; 2 Physiology and Ultrasound Laboratory in Science and Exercise, School of Human Movement and Nutrition Sciences, The University of Queensland, Level 2, Connell Building (26), St Lucia, Queensland 4072 Australia; 3 Centre for Research on Exercise, Physical Activity and Health, School of Human Movement and Nutrition Sciences, The University of Queensland, Level 2, Connell Building (26), St Lucia, Queensland 4072 Australia; 4 School of Public Health, The University of Queensland, Herston, Queensland 4006 Australia; 5 Mater Research Institute, Level 3, Aubigny Place, Raymond Terrace, South Brisbane, Queensland 4101 Australia

**Keywords:** Neoplasms, Cardiotoxicity, Pulse wave analysis, Carotid intima-media thickness, Exercise physiology, Physical activity

## Abstract

**Purpose:**

Cancer treatments exert vascular toxic effects that can lead to the development of cardiovascular disease. Exercise training has the potential to prevent or reduce cancer treatment–induced damage to vascular structure and function. This systematic review with meta-analyses aimed to determine the isolated effects of exercise training on vascular outcomes in people with cancer.

**Methods:**

Seven electronic databases were searched on 20 September 2021 to identify randomised controlled trials, quasi-randomised trials, pilot and cohort studies. Included studies implemented a structured exercise intervention and assessed vascular structure and/or function in people during or following cancer treatment. Meta-analyses examined the effects of exercise training on endothelial function (via brachial artery flow-mediated dilation) and arterial stiffness (via pulse wave velocity). Methodological quality was assessed using the Cochrane Quality Assessment tool and modified Newcastle-Ottawa Quality Appraisal tool. Grading of Recommendations, Assessment, Development and Evaluations framework was used to assess the certainty of evidence.

**Results:**

Ten studies (discussed across 11 articles) met the inclusion criteria. Methodological quality of the included studies was moderate (71% average). Exercise improved vascular function when compared to control (standardised mean difference = 0.34, 95% CI (0.01, 0.67); *p* = 0.044: studies = 5, participants = 171), but not pulse wave velocity (standardised mean difference = − 0.64, 95% CI (− 1.29, 0.02); *p* = 0.056: studies = 4, participants = 333). The certainty of evidence was moderate for flow-mediated dilation and low for pulse wave velocity.

**Conclusions:**

Compared to usual care, exercise training significantly improves flow-mediated dilation (endothelial function) but not pulse wave analysis, in people treated for cancer.

**Implications for Cancer Survivors:**

Exercise may improve vascular health in individuals during and following cancer treatment.

**Supplementary Information:**

The online version contains supplementary material available at 10.1007/s11764-023-01372-7.

## Introduction

In 2020, approximately 19.3 million individuals worldwide were diagnosed with cancer [1]. Whilst 5-year survival rates for several cancers have improved in many developed countries [2, 3], cardiovascular damage induced by cancer treatments [4] means cardiovascular disease (CVD) is now a leading cause of late morbidity and mortality in people with cancer [5, 6]. The vasculature represents a novel target for the early detection and prevention of CVD [7]. Endothelial dysfunction and arterial stiffness are important components of vascular dysfunction, which often precede overt CVD development [8–11]; are independent of traditional risk factors for CVD risk [8, 11, 12]; and predict CVD risk [10, 13]. Gold standard assessments of vascular dysfunction include brachial artery flow-mediated dilation (FMD) as a measure of endothelial function, pulse wave velocity (PWV) as a measure of arterial stiffness and carotid artery intima-media thickness (cIMT) as another measure of vascular structure.

Endothelial dysfunction and arterial stiffening are more pronounced in people with cancer compared to healthy individuals during cancer treatment [14, 15] and are worse following treatment [14–17]. Declines in FMD are also associated with detrimental changes to cardiac function during cancer treatment [18]. In women with breast cancer treated with anthracycline chemotherapy, FMD is significantly associated with changes in left ventricular ejection fraction, a traditional marker of cancer treatment–related cardiotoxicity [14]. Markers of early changes to vascular structure and function, such as FMD and PWV, could potentially be used to detect and prevent overt CVD, prior to irreversible dysfunction to organs such as the heart and brain.

Systematic reviews in cancer populations indicate exercise training is effective for improving holistic (i.e. cardiorespiratory fitness) [19, 20] and less specific (i.e. resting heart rate and peripheral blood pressure) [19, 21–23] indicators of cardiovascular health. Exercise also consistently demonstrates protective effects on cardiovascular health, including endothelial function [24, 25] and arterial stiffness [26], in apparently healthy adults and those with CVD. In people with cancer, a meta-analysis by Beaudry and colleagues [27] reported multi-modal lifestyle interventions, including exercise with or without dietary advice, improved endothelial function (standardised mean difference (SMD) = 0.65, 95% confidence intervals (95% CI) (0.33, 0.96): *I*^2^ = 0.00%) [27]. However, the assessment and interpretation of exercise-induced changes of these outcomes was confounded by the multi-modal interventions and minimal evaluation of the exercise dose variables (i.e. exercise intervention frequency, intensity, time and type (FITT) principles) of the included studies. Several published studies have since investigated the isolated effects of exercise on endothelial function and arterial stiffness in people with cancer [28–33].

Therefore, the aim of this paper is to systematically review and, where appropriate, meta-analyse the available literature to determine the isolated effects of exercise training interventions on vascular structure and function in people undergoing cancer treatment, or who have been previously treated for cancer.

## Methods

### Literature search

This review was reported in accordance with the Preferred Reporting Items for Systematic Reviews and Meta-Analyses (PRISMA) 2020 statement [34]. An extensive systematic search of seven databases (PubMed, Scopus, Web of Science, Embase, Cumulative Index to Nursing and Allied Health Literature (via EBSCOhost), MEDLINE (via EBSCOhost) and the Physiotherapy Evidence Database) was completed for articles up to 20 September 2021. Searches were performed for MESH terms (for PubMed) and free-text terms relating to cancer, AND-combination exercise, AND-combination vascular health; a full list of search terms for each database is provided in Online Resource 1. Free-text terms in titles relating to non-human trials were excluded. No additional articles were identified through screening the reference lists of articles included in full-text review.

### Inclusion and exclusion criteria

The Population, Intervention, Comparison, Outcomes and Study design (PICOS) framework was used to define the inclusion criteria. The population included children, adolescents or adults who had been diagnosed with any histologically confirmed cancer at any point during their lifetime. Participants could be undergoing or have completed any form of cancer treatment (e.g. chemotherapy, hormone therapy, surgery) at the time of the intervention. Any structured exercise intervention (describing mode, frequency, intensity, time and supervision level) was eligible, but could not include any additional allied health intervention (e.g. nutritional counselling). The control group could include usual care, wait list/delayed care or a comparison group of different or lesser exercise dose. For this review, if articles included non-cancer participants as a control/comparison (CON) group, the CON group’s data were not included in analyses and only within-group data from cancer group/s were analysed. Outcome measures of vascular function or structure (e.g. FMD, PWV, cIMT and pulse wave analysis (PWA) outcomes [i.e. central augmentation index, central augmentation pressure, central blood pressures]) were included. Articles that only reported peripheral blood pressure (or any derivative) and/or heart rate (resting, maximum) as measures of vascular health were excluded as these outcomes have been reviewed previously [19, 21, 35]. Most study designs were eligible, excluding case studies, cross-sectional studies and conference abstracts. Only articles including human trials published in peer-reviewed journals in the English language were included.

### Data extraction

Screening of titles and abstracts for each record was performed by two authors (N. K. V. and C. R. A. or Y. M.) using Covidence (Covidence systematic review software; Veritas Health Innovation, Melbourne, Australia). Duplicate records were automatically removed via Covidence prior to screening. PDF copies of full-text records were independently reviewed by two authors (N. K. V. and T. L. S. or Y. M.), with an arbitrator resolving any disagreements (T. G. B.). Reasons for exclusion of full-text records are provided in Online Resource 2. Data were manually extracted from full-text PDFs by two authors (N. K. V. and T. L. S. or Y. M.). Baseline and post-intervention timepoints were selected for data extraction. No missing data were present, and no authors were required to be contacted. Feasibility outcomes included study average monthly recruitment rate, attendance, adherence, dropout and safety of the intervention. Safety was assessed as rates of adverse events (AEs) and serious AEs which the authors directly attributed to the exercise intervention. For this review, AEs were defined as any non-serious event that negatively impacted participant health. Serious AEs were any AE deemed life-threatening, resulting in hospitalisation, permanent disability and/or death [36].

### Statistical analyses

Based on the current recommendations for the accurate conduct of systematic reviews with meta-analyses [37], the effect of exercise on FMD and PWV was evaluated using the DerSimonian-Laird random-effects model [38]. Pre- and post-study mean and standard deviation data were extracted for each group and transformed into change scores for analyses. Only exercise versus inactive control comparisons were considered. To avoid double-counting participants in Toohey et al. [32], the ‘shared’ group was split into two groups with smaller sample sizes and included two (reasonably independent) comparisons [39]. Meta-analyses were conducted by G. I. M. using Stata 16.1 (StataCorp. 2019, *Stata Statistical Software: Release 16*; StataCorp LLC, College Station, TX, USA), including the calculation of SMDs and 95% CI. The *Q* test was used to assess heterogeneity and the *I*^2^ measure of inconsistency to evaluate between-study variability. *p* values of < 0.05 were considered statistically significant. A qualitative review is presented for PWA and cIMT due to the lack of usual care comparator groups and an insufficient number of studies including these outcomes.

### Quality assessment

Each article was independently assessed for methodological quality by two authors (N. K. V. and T. L. S. or Y. M.), with an arbitrator resolving any disagreements (T. G. B). The Cochrane Quality Assessment tool [40] was used to determine the methodological quality of RCTs, controlled trials, comparative studies and non-controlled trials. The Modified Newcastle-Ottawa Quality Appraisal tool (ModNOS) [41] was used to assess the quality of cohort studies. A quality score for each article, represented as a percentage, was then calculated by dividing the total number of points scored by an article by the total number of questions/points for the given tool. Studies were not excluded based on their bias assessment.

### Certainty of evidence

For outcomes included in meta-analyses, certainty of the evidence was assessed using the Grading of Recommendations Assessment, Development and Evaluation (GRADE) framework [42] by two independent reviewers (N. K. V. and Y. M.). GRADEpro Guideline Development Tool (GDT) software (McMaster University and Evidence Prime) and its guidance on thresholds for each domain were used to conduct the assessment. There were no disagreements, and an arbitrator was not used.

## Results

### Study characteristics

**Fig. 1:**
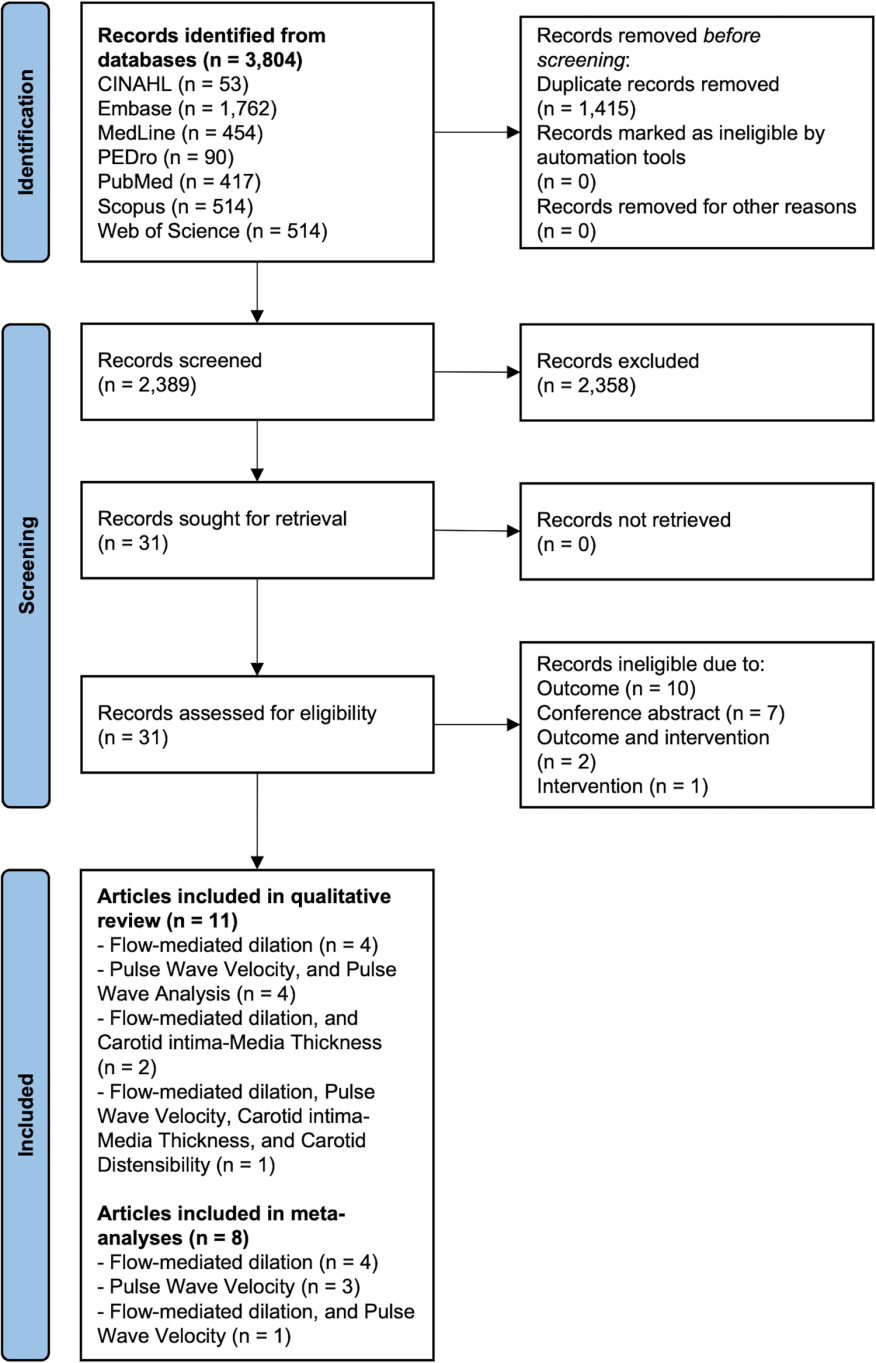
PRISMA flow diagram. Adapted from Page et al., [34]. *CINAHL* Cumulative Index to Nursing and Allied Health Literature, *PEDro* Physiotherapy Evidence Database

**Fig. 2:**
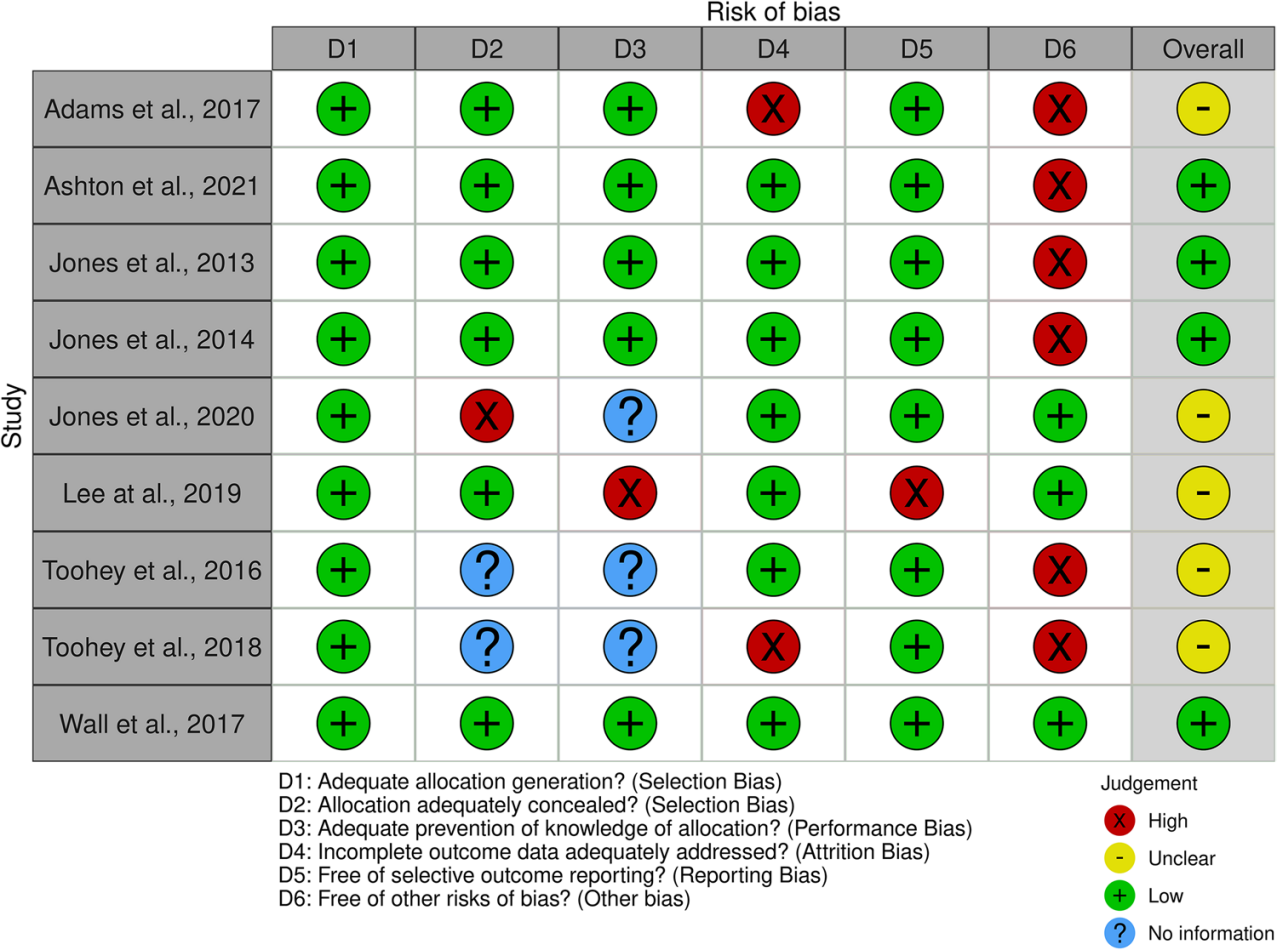
Risk of bias traffic light plot

A total of 3804 records were identified through database searching (Fig. 1). Following title and abstract screening, a total of 31 records underwent independent full-text review. Reasons for record exclusion are provided in Fig. 1 and Online Resource 2. A total of 11 articles, reporting on 10 independent studies/interventions, were included in the final analysis. Of the 10 included studies, one was a three-arm RCT (presented as such in one article [32] and as a two-arm randomised pilot study in another [43]), five were two-arm RCTs [28, 29, 33, 44, 45], one was a quasi-randomised trial [31], one was a two-arm randomised pilot study [30] and two were classified as cohort studies for the purpose of this review [46, 47]. Methodological quality scores for the Cochrane Quality Assessment tool and ModNOS ranged from 33% [32] to 100% [33] (Online Resource 3), with an average of 71% (Fig. 2).

### Participant characteristics

Sample sizes ranged from 13 [47] to 97 [33] participants, with a cumulative sample of 444 participants across the 10 independent studies (Table 1). Participants ranged in age from adolescents to adults (range = 16 [47] to 78 [33] years), with a total of 268 male and 176 female participants. Studies included those actively undergoing treatment during the study period and/or those who had finished treatment for breast [30, 31, 44], prostate [29, 33, 45], testicular [28], childhood [46, 47] or mixed cancer populations [32, 43]. Three studies included participants actively on treatment throughout the study period [30, 33, 44]. Two reported participants actively undergoing chemotherapy [30, 44] and another undergoing hormone therapy with or without radiotherapy [33]. The remaining studies included participants who had finished one or more treatments (e.g. surgery, radiotherapy, chemotherapy and/or hormone therapy) prior to study initiation [28, 29, 31, 32, 43, 45–47]. Time since treatment for participants in these studies ranged from 8 weeks [31] to ~ 19 years [47]. Comprehensive exercise intervention details for each of the 11 articles, including FITT principles and supervision level, are provided in Table 1. Eight studies provided sufficient data for meta-analyses of FMD (*N* = 5) [28–30, 44, 45] and/or PWV (*N* = 4) [28, 31–33] outcomes.

**Table 1:**
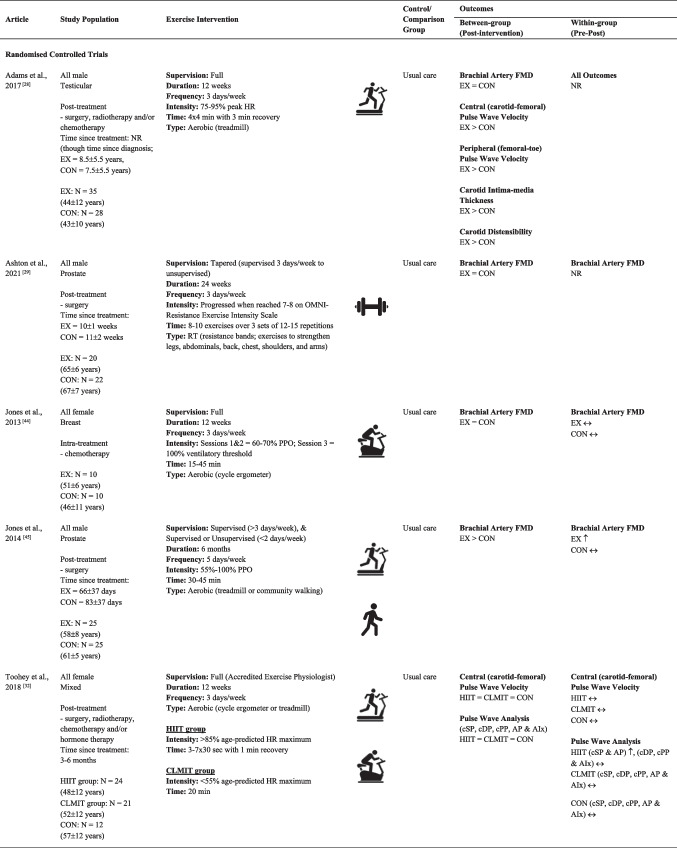

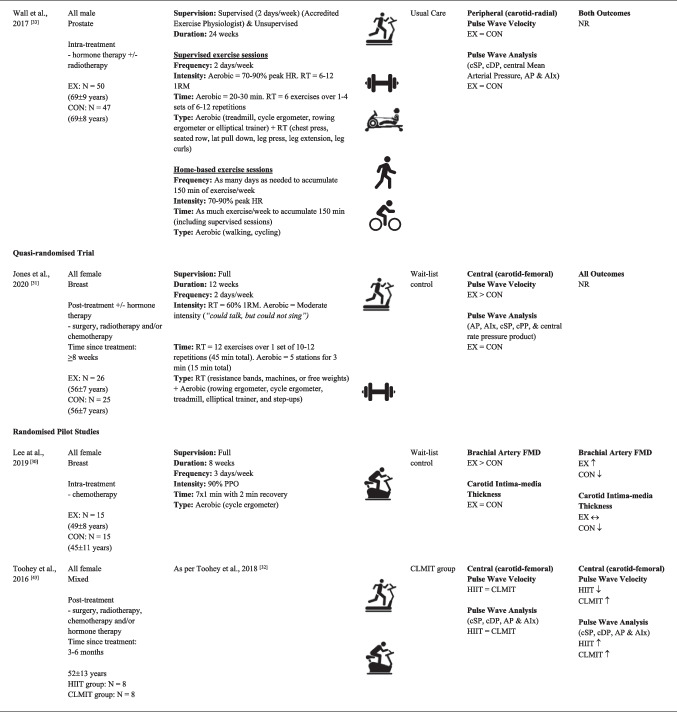

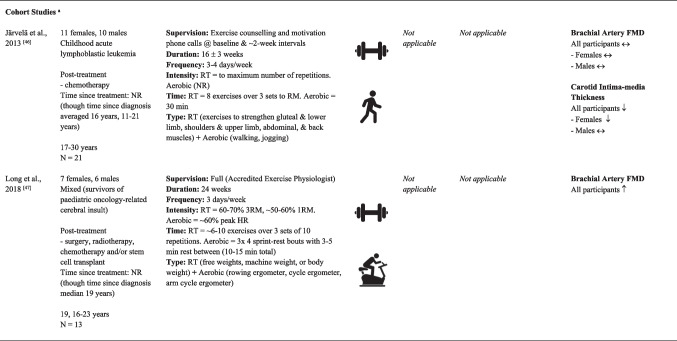
Study characteristics of included articles

EX > CON = significant between-group difference favouring intervention group; EX < CON = significant between-group difference favouring control/comparison group; EX = CON = no significant between-group difference; ↑ = significant within-group improvement in outcome/s; ↓ = significant within-group worsening in outcome/s; ↔ = no change in outcome/s

*AIx* augmentation index, *AP* augmentation pressure, *FMD* endothelial-dependent flow-mediated dilation, *cDP* central diastolic pressure, *CLMIT* continuous low-to-moderate intensity training, *CON* control/comparator group, *cPP* central pulse pressure, *cSP* central systolic pressure, *EX* exercise intervention group, *HIIT* high-intensity interval training, *HR* heart rate, *min* minutes, *NR* not reported, *PPO* peak power output, *RM* repetition maximum, *RT* resistance training

^a^Between- and within-group level data from control/comparison groups comprising a non-cancer population were treated as cohort studies

Average monthly recruitment rate was able to be calculated in eight of the 11 included articles [28–30, 32, 33, 43–45], ranging from less than one participant [44] to seven participants [28] per month (average three participants randomised per month). Ten of the 11 included articles [28–33, 43–45, 47] reported attendance to the supervised exercise intervention, ranging from 69% [33] to 100% [47] and averaging 86%. Exercise prescription adherence was reported in only four of the 11 articles [28, 44, 45, 47], ranging from 66% [44] to 101% [28] and averaging 87%. All articles reported participant dropout, with most reporting reasons for dropout (Table 2) [30–33, 43–47]. Study dropout ranged from 0% [30, 43, 46, 47] to 32% [32], averaging 8%. Overall, the dropout rate was higher for CON groups (total participants, *N* = 36) compared with intervention groups (total participants, *N* = 17). Nine of the 11 articles [28–33, 43–45, 47] reported AEs and serious AEs, with a total of 131 independent AEs [29, 44, 45] and no serious AEs. A total of 129 non-serious independent AEs were reported in a single article [45], with the majority being attributed to exercise training–induced leg cramps and back pain that required modification or early cessation of exercise training sessions.

**Table 2:** Feasibility outcomes of included articles

Article	Attendance ^a^	Adherence ^b^	Dropout rates and reason for dropout	Adverse events	Serious adverse events	Average monthly recruitment rate ^c^
Adams et al., 2017 [28]	99%	98% (works periods) 103% (active recovery periods)	Reason NR: *N* = 1 (CON)	Nil	Nil	7 participants
Ashton et al., 2021 [29]	94% (months 0–3) 78% (months 3–6)	NR	Uncontactable: *N* = 2 (1 EX, 1 CON) Back injury (unrelated to project): *N* = 1 (EX) Hernia (unrelated to project): *N* = 1 (CON) Family bereavement: *N* = 1 (CON)	Rotator cuff injury, resolved: *N* = 1 (EX)	Nil	4 participants
Järvelä et al., 2013 [46]	Not collected	Not collected	Nil	NR	NR	NR
Jones et al., 2013 [44]	82%	66%	Deep vein thrombosis + pulmonary embolism: *N* = 1 (EX)	Unexplained leg pain, resolved: *N* = 1 (EX)	Nil	Less than 1 participant
Jones et al., 2014 [45]	83% (supervised) 72% (home-based)	79% (supervised) NR (home-based)	Lost to follow-up: *N* = 4 (2 EX, 2 CON)	*N* = 129 Training-induced leg cramps = 55% Training-induced back pain = 26%	Nil	2 participants
Jones et al., 2020 [31]	92%	NR	Ankle injury (unrelated to project): *N* = 1 (CON) Moved away: *N* = 1 (CON) Other: *N* = 1 (CON)	Nil	Nil	NR
Lee at al., 2019 [30]	82%	NR	Nil	Nil	Nil	3 participants
Long et al., 2018 [47]	100% (for participants included in final analyses)	≥ 90% (for participants included in final analyses)	Non-compliance to intervention (not included in final analyses): *N* = 5 Relapse: *N* = 2	Nil	Nil	NR
Toohey et al., 2016 [43] ^d^	94% (LVHIIT & CLMIT)	NR	Nil	Nil	Nil	2 participants
Toohey et al., 2018 [32] ^d^	92% (HIIT & CLMIT)	NR	Unmotivated: *N* = 5 (1 CLMIT, 4 CON) Reason NR: *N* = 5 (1 CLMIT, 4 CON) Lost to follow-up: *N* = 3 (1 CLMIT, 2 CON) Moved away: *N* = 2 (1 CLMIT, 1 CON) Change in employment: *N* = 1 (HIIT) Injury (unrelated to project): *N* = 1 (CON) Travelled overseas: *N* = 1 (CON)	Nil	Nil	4 participants
Wall et al., 2017 [33]	69% (supervised) NR (home-based)	NR (both)	No longer interested: *N* = 5 (1 EX, 4 CON) Other: *N* = 4 (2 EX, 2 CON) Injury: *N* = 3 (1 EX, *N* = 2 CON) Ineligible: *N* = 3 (2 EX, 1 CON) Health: *N* = 2 (1 EX, 1 CON) Moved away: *N* = 1 (CON) Died: *N* = 1 (CON) Lost to follow-up: *N* = 1 (CON) Personal reasons: *N* = 1 (CON)	Nil	Nil	3 participants

*CLMIT* continuous low-to-moderate intensity training, *CON* control/comparator group, *EX* exercise intervention group, *HIIT* high-intensity interval training, *NR* not reported

^a^The number of sessions completed by the exercise intervention group, compared to the number of planned sessions

^b^The percentage of exercise sessions performed at the prescribed intensity and/or duration

^c^The average number of participants randomised each month during the study period

^d^Outcomes from the three-arm RCT (presented across two articles) separated due to further participant recruitment from 2016 [43] to 2018 [32]

### Efficacy of exercise interventions

Seven studies [28–30, 44–47] assessed exercise-induced changes in brachial artery FMD. Four studies (discussed across five articles) assessed exercise-induced changes in arterial stiffness via central and/or peripheral PWV, compared with usual care and/or lower-intensity training (total participants, *N* = 333) [28, 31–33, 43].

#### Meta-analysed data

**Fig. 3:**
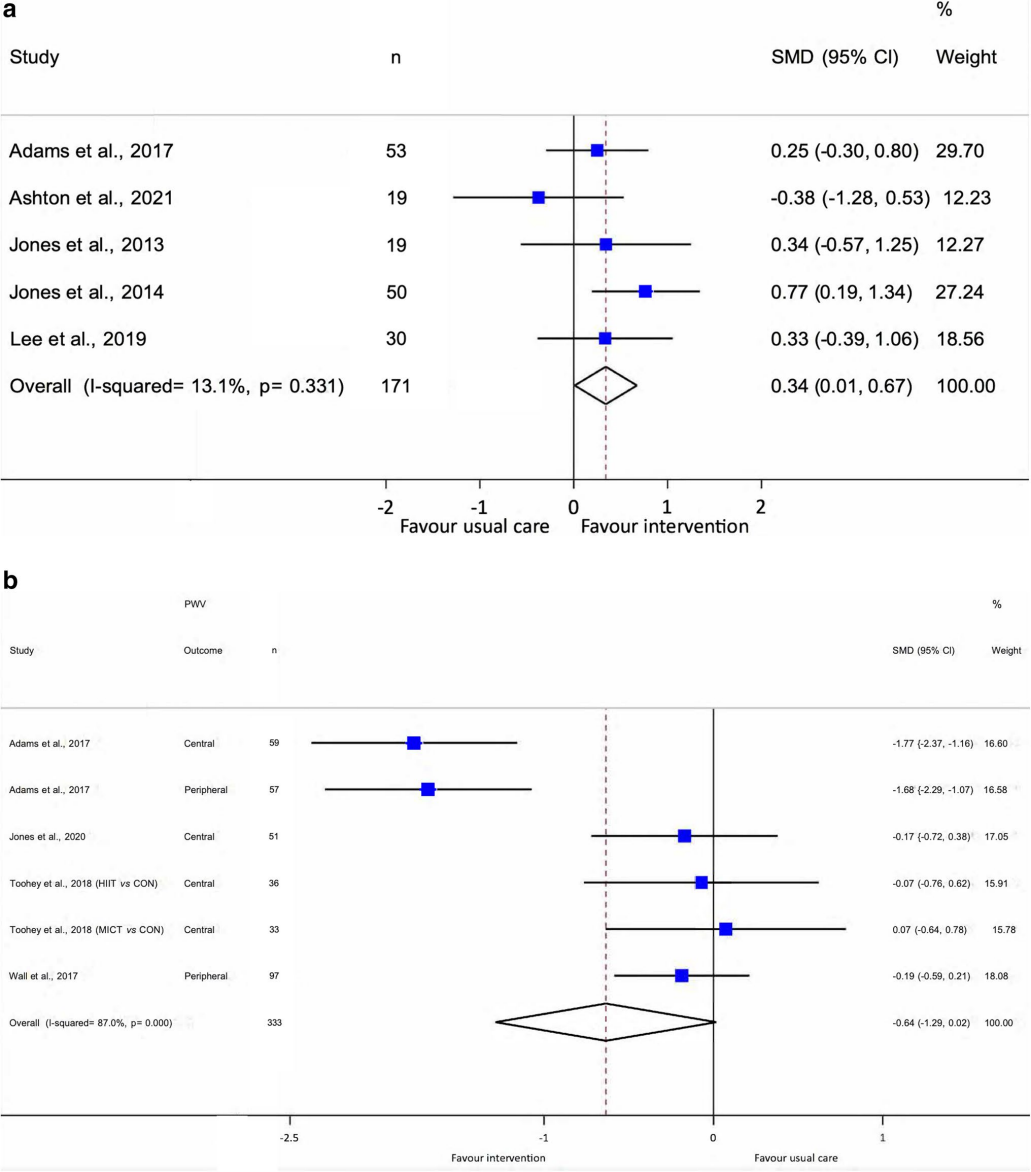
Forest plots of flow-mediated dilation and pulse wave velocity in usual care vs. exercise intervention. Data depicts the standardised mean difference and 95% CI for **a** flow-mediated dilation and **b** pulse wave velocity (combined central and peripheral) in individual studies and pooled estimates. *CI* confidence intervals, *CON* control (usual care) group, *HIIT* high-intensity interval training, *MICT* continuous low-to-moderate intensity training, *PWV* pulse wave velocity, *SMD* standardised mean difference

In the FMD meta-analysis (studies, *N* = 5; total participants, *N* = 171) [28–30, 44, 45], FMD% was significantly improved in two [30, 45] exercise interventions which used high-intensity exercise training compared with usual care. Overall, we have a moderate certainty of evidence that the SMD for FMD% significantly favours exercise compared to usual care (0.34, 95% CI (0.01, 0.67); *p* = 0.044: *Q* test for heterogeneity, *p* = 0.331, *I*^2^ = 13.1%) (Fig. 3; Online Resource 4).

In the PWV meta-analysis, two of the four studies observed significant between-group improvements in central PWV favouring the exercise group [28, 31], with one also observing a significant improvement in peripheral PWV [28]. Overall, there is a low certainty of evidence for no effect of exercise on arterial stiffness (as assessed by combined PWV) (SMD = − 0.64, 95% CI (− 1.29, 0.02); *p* = 0.056: *Q* test for heterogeneity, *p* < 0.001, *I*^2^ = 87.0%) (Fig. 3; Online Resource 4). SMDs according to PWV sub-group (central and peripheral) were also investigated (Online Resource 5). The sub-group SMDs also showed no effect of exercise on central (− 0.49, 95% CI (− 1.35, 0.37); *p* = 0.262: *Q* test for heterogeneity, *p* < 0.001, *I*^2^ = 86.4%) or peripheral (− 0.92, 95% CI (− 2.38, 0.55); *p* = 0.221: *Q* test for heterogeneity, *p* < 0.001, *I*^2^ = 93.8%) PWV.

Using the GRADE assessment tool (Table 3 and Online Resource 6), we observed a moderate certainty of evidence for the effect of exercise on FMD, which was downgraded owing to a small total sample size. We observed low certainty of evidence for the effect of exercise on PWV (combined), which was downgraded owing to some concerns with CIs, and high heterogeneity.

**Table 3:** Summary of assessment of certainty of evidence (GRADE) for outcomes

Outcome	Number of participants (studies)	Standardised mean difference (95% CI)	*I*^2^ (%)	Certainty of evidence (GRADE)
Flow-mediated dilation	171 (5 RCTs)	0.34 (0.01, 0.67) higher	13.1	Moderate ^a^
Pulse wave velocity	333 (4 RCTs)	− 0.64 (− 1.29, 0.02) lower	87.0	Low ^b^

*CI* confidence intervals, *GRADE* Grading of Recommendations Assessment, Development and Evaluation, *RCTs* randomised controlled trials

^a^Small total sample size

^b^Some concerns with CIs, and high heterogeneity

#### Non-meta-analysed data

For the FMD outcome, the two cohort studies in post-treatment adolescent and adult survivors of childhood cancer reported mixed findings. A 24-week moderate-intensity aerobic and resistance training (RT) combined circuit intervention ~ 19 years after diagnosis and treatment for paediatric oncology–related cerebral insult resulted in a moderate improvement in FMD% (*p* = 0.029, *d* = 0.63) [47]. However, a similar effect was not observed in a ~ 16-week near-maximal-to-maximal-intensity combined resistance and continuous aerobic intervention in adolescents and adults ~ 16 years after diagnosis of childhood acute lymphoblastic leukaemia [46]. For the PWV outcome, the one comparative interventional study in this review (discussed across two articles) compared 12 weeks of high-intensity interval training (HIIT) with continuous low-to-moderate-intensity training and found no significant difference between groups for central PWV in a mixed cancer population [32, 43].

Exercise-induced changes in cIMT were assessed in three studies [28, 30, 46], with one including a further assessment of carotid artery distensibility [28]. The 12-week moderate-to-high-intensity exercise study in men after treatment for testicular cancer observed significant between-group improvements for average cIMT (− 0.06 mm; *p* < 0.001), maximum cIMT (− 0.08 mm; *p* < 0.001) and carotid distensibility (+ 1.54 10^−3^/kPa; *p* = 0.049), favouring the exercise compared to the CON group [28]. A significant within-group improvement in cIMT (− 2.8% relative change; *p* = 0.02, *d* = − 0.61) was observed following the ~ 16-week combined intervention in those living beyond childhood acute lymphoblastic leukaemia. Sub-group analyses identified the significant improvement in cIMT was observed in females (− 2.3%; *p* = 0.04, *d* = − 0.55) but not in males (− 3.4%; *p* = 0.11, *d* = − 0.65) [46]. However, a shorter 8-week interval intervention performed at a similar higher intensity in women undergoing chemotherapy for breast cancer did not observe a significant between-group difference in cIMT (*p* = 0.23) [30].

Three studies [31–33, 43] reported changes in central aortic wave reflection characteristics via PWA. One (discussed across two articles) [32, 43] compared 12 weeks of low-volume HIIT with continuous low-to-moderate-intensity training, with or without usual care, in a mixed cancer survivor population. Another study included a 12-week moderate-intensity combined resistance and continuous aerobic training intervention in women living beyond breast cancer [31], and the other a 6-month vigorous-intensity combined resistance and continuous aerobic training intervention in men undergoing hormone therapy for prostate cancer [33]. None of these studies reported significant improvements in PWA outcomes (central augmentation index, central augmentation pressure, central blood pressures), when compared with usual care or lower-intensity exercise, in adults undergoing or who had finished treatment for cancer [31–33, 43].

## Discussion

This is the first systematic review with meta-analysis to report the isolated effects of exercise training on markers of vascular function and structure in people undergoing or following treatment for cancer. Ten moderate-quality studies (discussed across 11 articles), comprising 444 participants, were included in the analyses. Meta-analyses revealed that exercise training significantly improves endothelial function (FMD) compared to usual care. Exercise training trended to improve arterial stiffness (PWV) compared to usual care. Preliminary evidence suggests exercise positively influences cIMT, but not PWA outcomes.

Brachial artery FMD provides an indication of nitric oxide–mediated endothelial-dependent vascular function and is an independent predictor of future cardiovascular events [11]. As all studies in this review utilised forearm occlusion [28–30, 44–47], our findings represent nitric oxide–mediated endothelial-dependent vascular function. Our meta-analysis found exercise training in people with cancer has a significant positive influence on FMD (SMD = 0.34, 95% CI (0.01, 0.67); *p* = 0.044. Although significant, this was less than previously reported on combined FMD and reactive hyperaemia outcomes in the 2018 meta-analysis of lifestyle interventions (0.34 vs. 0.65 [27]). A key point of difference might be the inclusion of a higher number of studies in the current review and that, in our analysis, exercise was delivered both during [30, 44] and after [28, 29, 45] cancer treatment. Differing treatment and cancer types likely have significant effects on exercise-induced changes in FMD. Two of the five studies included in this FMD meta-analysis comprised adults undergoing anthracycline-containing chemotherapy regimens [30, 44]. Anthracyclines are known to be highly cardiotoxic [4], and these women [30, 44] likely experienced greater cardiovascular damage than those in the two studies to include men with prostate cancer who had only undergone surgery [29, 45]. Furthermore, the type of cancer must be considered. The median age at diagnosis of prostate cancer is greater than that of breast cancer (67 vs. 63 years, respectively [48, 49]), and CVD outcomes in cancer populations appear to worsen with increasing age [50]. Meanwhile, the study that included higher-intensity aerobic exercise training in adults undergoing highly cardiotoxic anthracycline-containing chemotherapy observed a significant between-group difference in FMD, favouring the exercise group [30]. High-intensity interval training has been found to be superior to moderate-intensity continuous training for improving endothelial function in preclinical [51] and clinical [52] studies. This is likely related to HIIT-mediated increases in nitric oxide bioavailability, increased expression of antioxidant enzymes, reductions in the expression of proinflammatory molecules and/or increases in the number of endothelial progenitor cells [52].

The duration of the exercise-induced haemodynamic stimulus could play a greater role in improving FMD than intensity for adults and adolescents who have completed cancer treatment [28, 45–47]. Studies in this review with longer intervention durations [47] or higher numbers of weekly exercise sessions [45] experienced superior improvements in FMD%, compared to those with a lower total dose [28, 46]. Improvements in endothelial function are induced by the repetitive increases in arterial blood flow and shear stress with acute bouts of aerobic exercise [53]. Longer-term exercise training and repeated bouts of exercise expose the vasculature to greater amounts of shear stress, leading to reductions in inflammation and oxidative stress [54], and could subsequently slow deterioration of the vasculature following treatment. Indeed, this is consistent with a recent meta-analysis that demonstrated longer exercise interventions (≥ 2 years) can elicit superior improvements in FMD% compared to sedentary controls [55]. Collectively, preliminary findings suggest higher-intensity exercise could be more beneficial for maintaining or improving FMD in adults currently undergoing cardiotoxic treatments [30, 44], whilst adherence to longer-term exercise is likely important for beneficial effects on FMD in people following treatment for cancer [28, 45–47]. This notion is important given the delayed cardiotoxic effects of cancer treatment. Further research is required to confirm these preliminary findings. Identifying the optimal dose and timing of exercise to elicit improvements in FMD would provide an opportunity to mitigate CVD risk and reduce long-term cardiotoxicity [56–58].

This review suggests exercise training may lower arterial stiffness (as assessed by PWV (combined)) compared to usual care (SMD = − 0.64, 95% CI (− 1.29, 0.02)) in cancer populations, though this finding was not statistically significant (*p* = 0.056). This overall effect is similar in magnitude to the exercise-induced reductions in PWV previously observed in healthy adults (− 0.67, 95% CI (− 0.97, − 0.38); *p* < 0.00001: *I*^2^ = 89.0%) [59]. The two 12-week studies that assessed PWV in the current review prescribed aerobic interval training at moderate [31] or vigorous-to-near-maximal/maximal intensity [28] and observed significant improvements in central PWV compared to usual care in people following cancer treatment. This suggests that 12 weeks of interval aerobic training (aerobic-only [28] or concurrently with RT [31]) is effective in improving central PWV, regardless of the intensity prescribed. Furthermore, the intervention by Toohey and colleagues [32, 43] compared the effects of aerobic HIIT to continuous low-to-moderate intensity training [43], with or without usual care [32], and observed no significant difference between groups post-study for central PWV in both articles. This agrees with findings in mixed adult populations, where no significant difference in the change in PWV has been observed between HIIT and moderate intensity continuous training [60]. Collectively, 12 weeks of moderate- or higher-intensity interval training (aerobic-only [28] or concurrent with RT [31]) could reduce central PWV, though, currently, the evidence suggests the overall effect of exercise is minimal and non-significant.

This review identified preliminary evidence that cIMT improves with 12 weeks [28] to 26 weeks [46], but not 8 weeks [30], of exercise training. This might be explained by functional preceding structural changes in the vasculature with exercise training [53]. Meanwhile, no changes in any PWA outcomes were observed in any study, including augmentation index and central blood pressure [31–33, 43]. This is despite peripheral blood pressure being significantly improved with exercise training in breast cancer populations [21, 22], suggesting peripheral changes may precede central blood pressure changes [24]. Further research is required to confirm the effects of exercise training on cIMT and central haemodynamics in this population.

The recent Exercise and Sport Science Australia [61], American College of Sports Medicine [62] and Clinical Oncology Society of Australia [63] position statements highlight the need for multi-modal moderate-to-high intensity exercise training to improve the physical and psychosocial health of people undergoing treatment and those who have finished treatment. Currently, there is no guideline for improving CVD risk in people during and following cancer treatment. This review, and evidence in healthy and clinical groups [53], supports the use of aerobic exercise for targeting improvements in vascular health. However, the optimal dose required, particularly during and after treatment for cancer, is unknown. Randomised controlled trials powered for CVD outcomes, including endothelial function and arterial stiffness, are required to confirm the efficacy of structured and tailored exercise interventions to mitigate and prevent CVD risk in this population. This is a vital piece of evidence required to inform the exercise guidelines for individuals with cancer and their practitioners interested in improving vascular health during and following treatment for cancer.

### Limitations

Our findings add to the growing body of evidence that structured exercise training appears safe during and after treatment for cancer [61, 62]. However, the low study average monthly recruitment rates significantly affect the feasibility of such interventions in this common clinical population. Varied exercise intervention characteristics and the low number of studies (*N* = 4) reporting adherence to the prescribed interventions precluded identification of the optimal ‘dose’ to enhance or maintain the cardiovascular health of people treated for cancer. The more accurate reporting of feasibility outcomes (i.e. FITT principles, intervention adherence) would enable researchers in the future to guide the development and implementation of larger RCTs to determine the optimal exercise dose to prevent and/or mitigate CVD in people treated for cancer. The findings of this review should be interpreted with caution due to the small number of studies, large heterogeneity in study populations and exercise intervention designs, the large confidence intervals of the pooled effects and the moderate methodological quality of the articles. Due to the limited availability of evidence, adult and childhood cancer survivor groups, as well as individuals with differing treatment types and times since treatment, were pooled together in quantitative and qualitative analyses. It was not possible to isolate the effects of exercise in these different demographic and clinical outcomes, and this should be a priority for future research. Quality of the assessments of outcome measures also varied across the studies. Most measures included in this review are heavily dependent on tester skill and experience, but no indication of intra- or inter-tester reliability was provided. It is also plausible that some of the studies in this review were underpowered to detect between-group differences in the reported vascular health outcomes [64]. Typical limitations of exercise oncology research were also present, such as the absence of older adults and rare or more advanced cancers [65].

## Conclusions

This is the first review to investigate the isolated effects of exercise training on vascular health in individuals undergoing or who have finished treatment for cancer. Our findings suggest a moderate certainty of evidence that structured exercise training significantly improves endothelial function (FMD), and a low certainty of evidence for no effect on arterial stiffness (PWV). Preliminary evidence suggests exercise positively influences cIMT, but not PWA outcomes. Exercise-mediated improvements in FMD are likely enhanced by higher-intensity interventions in those actively on treatment, and longer-duration (> 12-week) interventions in those who have finished treatment. Improvements in vascular health with exercise training have the potential to prevent and/or mitigate future CVD in people treated for cancer. Exercise should be recommended for individuals during and following cancer treatment to improve vascular health.

### Supplementary information

Online Resource 1 Databases systematic search terms.

Online Resource 2 List of references excluded during full-text review and reason for exclusion.

Online Resource 3 Quality assessment tools risk-of-bias.

Online Resource 4 Meta-analyses funnel plots.

Online Resource 5 Forest plot of pulse wave velocity in usual care vs. exercise intervention according to pulse wave velocity sub-group (central and peripheral), and overall combined.

Online Resource 6 Individual domains and overall GRADE assessment.
