# Supplementary material for: Efficacy of exercise training for improving vascular dysfunction in people with cancer: a systematic review with meta-analyses
Source: J Cancer Surviv. 2023 Apr 20;18(4):1309–24. doi: 10.1007/s11764-023-01372-7 (PMC11324680; doi:10.1007/s11764-023-01372-7)
Supplement: Supplementary file 1 — Databases systematic search terms. [file 11764_2023_1372_MOESM1_ESM.pdf]

Online Resource 1 - Databases systematic search terms

PubMed

((("Neoplasms"[Mesh] OR "neoplasm" OR "cancer*" OR "carcinoma*" OR "malignan*") AND ("Exercise"[Mesh] OR "physical activity" OR "physical intervention" OR "physical fitness" OR "sport*" OR "intensity exercise" OR "weight train*" OR "resistance train*" OR "strength train*" OR "muscular strength" OR "aerobic" OR "endurance" OR "walk*" OR "jog" OR "jogging" OR "run" OR "running" OR "cycling" OR "swim*" OR "pilates" OR "yoga" OR "tai chi" OR "stretch*")) AND ("endothelial function" OR "endothelial dysfunction" OR "arterial function" OR "arterial dysfunction" OR "vascular function" OR "vascular dysfunction" OR "vascular health" OR "arterial health" OR "arterial stiffness" OR "macrovascular function" OR "microvascular function" OR "atherosclerosis" OR "flow mediated dilation" OR "flow-mediated dilation" OR "brachial artery reactivity" OR "carotid artery media thickness" OR "carotid intima-media thickness" OR "central blood pressure*" OR "carotid distensibility" OR "pulse wave analysis" OR "pulse wave velocity" OR "strain gauge plethysmography")) NOT ("rat"[Title] OR "rats"[Title] OR "mouse"[Title] OR "mice"[Title])

Scopus

(ALL ("Neoplasms" [mesh] OR "neoplasm" OR "cancer*" OR "carcinoma*" OR "malignan*") AND ALL ("Exercise" [mesh] OR "physical activity" OR "physical intervention" OR "physical fitness" OR "sport*" OR "intensity exercise" OR "weight train*" OR "resistance train*" OR "strength train*" OR "muscular strength" OR "aerobic" OR "endurance" OR "walk*" OR "jog" OR "jogging" OR "run" OR "running" OR "cycling" OR "Swim*" OR "pilates" OR "yoga" OR "tai chi" OR "stretch*") AND TITLE-ABS-KEY ("endothelial function" OR "endothelial dysfunction" OR "arterial function" OR "arterial dysfunction" OR "vascular function" OR "vascular dysfunction" OR "vascular health" OR "arterial health" OR "arterial stiffness" OR "macrovascular function" OR "microvascular function" OR "atherosclerosis" OR "flow mediated dilation" OR "flow-mediated dilation" OR "brachial artery reactivity" OR "carotid artery media thickness" OR "carotid intima-media thickness" OR "central blood pressure*" OR "carotid distensibility" OR "pulse wave analysis" OR "pulse wave velocity" OR "strain gauge plethysmography") AND NOT TITLE ("rat" OR "rats" OR "mouse" OR "mice"))

Web of Science

# 6	380	#5 NOT #4<br>*Indexes=SCI-EXPANDED, SSCI, A&HCI, CPCI-S, CPCI-SSH, BKCI-S, BKCI-SSH, ESCI, CCR-EXPANDED, IC Timespan=All years*
# 5	387	#3 AND #2 AND #1<br>*Indexes=SCI-EXPANDED, SSCI, A&HCI, CPCI-S, CPCI-SSH, BKCI-S, BKCI-SSH, ESCI, CCR-EXPANDED, IC Timespan=All years*
# 4	1,774,194	**TITLE:** ("rat" OR "rats" OR "mouse" OR "mice")<br>*Indexes=SCI-EXPANDED, SSCI, A&HCI, CPCI-S, CPCI-SSH, BKCI-S, BKCI-SSH, ESCI, CCR-EXPANDED, IC Timespan=All years*
# 3	270,388	**TOPIC:**<br>("endothelial function" OR "endothelial dysfunction" OR "arterial function" OR "arterial dysfunction" OR "vascular function" OR "vascular dysfunction" OR "vascular health" OR "arterial health" OR "arterial stiffness" OR "macrovascular function" OR "microvascular function" OR "atherosclerosis" OR "flow mediated dilation" OR "flow-mediated dilation" OR "brachial artery reactivity" OR "carotid artery media thickness" OR "carotid intima-media thickness" OR "central blood pressure*" OR "carotid distensibility" OR "pulse wave analysis" OR "pulse wave velocity" OR "strain gauge plethysmography")<br>*Indexes=SCI-EXPANDED, SSCI, A&HCI, CPCI-S, CPCI-SSH, BKCI-S, BKCI-SSH, ESCI, CCR-EXPANDED, IC Timespan=All years*
# 2	1,640,776	**TOPIC:**<br>("Exercise"[Mesh] OR "physical activity" OR "physical intervention" OR "physical fitness" OR "sport*" OR "intensity exercise" OR "weight train*" OR "resistance train*" OR "strength train*" OR "muscular strength" OR "aerobic" OR "endurance" OR "walk*" OR "jog" OR "jogging" OR "run" OR "running" OR "cycling" OR "swim*" OR "pilates" OR "yoga" OR "tai chi" OR "stretch*")<br>*Indexes=SCI-EXPANDED, SSCI, A&HCI, CPCI-S, CPCI-SSH, BKCI-S, BKCI-SSH, ESCI, CCR-EXPANDED, IC Timespan=All years*
# 1	3,371,016	**TOPIC:** ("Neoplasms"[Mesh] OR "neoplasm" OR "cancer*" OR "carcinoma*" OR "malignan*")<br>*Indexes=SCI-EXPANDED, SSCI, A&HCI, CPCI-S, CPCI-SSH, BKCI-S, BKCI-SSH, ESCI, CCR-EXPANDED, IC Timespan=All years*

Embase

No.	Query
**#5**	**#1** AND **#2** AND **#3** NOT **#4**
**#4**	**'rat'**:ti OR **'rats'**:ti OR **'mouse'**:ti OR **'mice'**:ti
**#3**	**'endothelial function'** OR **'endothelial dysfunction'** OR **'arterial function'** OR **'arterial dysfunction'** OR **'vascular function'** OR **'vascular dysfunction'** OR **'vascular health'** OR **'arterial health'** OR **'arterial stiffness'** OR **'macrovascular function'** OR **'microvascular function'** OR **'atherosclerosis'** OR **'flow mediated dilation'** OR **'flow-mediated dilation'** OR **'brachial artery reactivity'** OR **'carotid artery media thickness'** OR **'carotid intima-media thickness'** OR **'central blood pressure*'** OR **'carotid distensibility'** OR **'pulse wave analysis'** OR **'pulse wave velocity'** OR **'strain gauge plethysmography'**
**#2**	**'exercise'** OR **'physical activity'** OR **'physical intervention'** OR **'physical fitness'** OR **'sport*'** OR **'intensity exercise'** OR **'weight train*'** OR **'resistance train*'** OR **'strength train*'** OR **'muscular strength'** OR **'aerobic'** OR **'endurance'** OR **'walk*'** OR **'jog'** OR **'jogging'** OR **'run'** OR **'running'** OR **'cycling'** OR **'swim*'** OR **'pilates'** OR **'yoga'** OR **'tai chi'** OR **'stretch*'**
**#1**	**'neoplasms'** OR **'neoplasm'** OR **'cancer*'** OR **'carcinoma*'** OR **'malignan*'**

Cumulative Index to Nursing and Allied Health Literature (via EBSCOhost)

("Neoplasms"[Mesh] OR "neoplasm" OR "cancer*" OR "carcinoma*" OR "malignan*") AND ("Exercise"[Mesh] OR "physical activity" OR "physical intervention" OR "physical fitness" OR "sport*" OR "intensity exercise" OR "weight train*" OR "resistance train*" OR "strength train*" OR "muscular strength" OR "aerobic" OR "endurance" OR "walk*" OR "jog" OR "jogging" OR "run" OR "running" OR "cycling" OR "swim*" OR "pilates" OR "yoga" OR "tai chi" OR "stretch*") AND ("endothelial function" OR "endothelial dysfunction" OR "arterial function" OR "arterial dysfunction" OR "vascular function" OR "vascular dysfunction" OR "vascular health" OR "arterial health" OR "arterial stiffness" OR "macrovascular function" OR "microvascular function" OR "atherosclerosis" OR "flow mediated dilation" OR "flow-mediated dilation" OR "brachial artery reactivity" OR "carotid artery media thickness" OR "carotid intima-media thickness" OR "central blood

pressure*" OR "carotid distensibility" OR "pulse wave analysis" OR "pulse wave velocity" OR "strain gauge plethysmography") NOT ("rat"[Title] OR "rats"[Title] OR "mouse"[Title] OR "mice"[Title])

MEDLINE (via EBSCOhost)

("Neoplasms" OR"neoplasm" OR "cancer*"OR "carcinoma*" OR"malignan*") AND ("Exercise" OR "physicalactivity" OR "physicalintervention" OR "physicalfitness" OR "sport*" OR"intensity exercise" OR"weight train*" OR"resistance train*" OR"strength train*" OR"muscular strength" OR"aerobic" OR "endurance"OR "walk*" OR "jog" OR“jogging” OR "run" OR“running” OR "cycling" OR"swim*" OR "pilates" OR"yoga" OR "tai chi" OR"stretch*") AND ("endothelial function" OR"endothelial dysfunction" OR"arterial function" OR"arterial dysfunction" OR"vascular function" OR"vascular dysfunction" OR"vascular health" OR“arterial health” OR "arterialstiffness" OR“macrovascular function”OR "microvascular function"OR "atherosclerosis" OR"flow mediated dilation" OR“flow-mediated dilation” OR"brachial artery reactivity"OR "carotid artery mediathickness" OR “carotidintima-media thickness” OR“central blood pressure*”OR “carotid distensibility”OR "pulse wave analysis"OR "pulse wave velocity"OR "strain gaugeplethysmography") NOT TI("rat" OR "rats" OR "mouse"OR "mice")

Physiotherapy Evidence Database

cancer AND exercise AND cardiovascular
