# Supplementary material for: Efficacy of exercise training for improving vascular dysfunction in people with cancer: a systematic review with meta-analyses
Source: J Cancer Surviv. 2023 Apr 20;18(4):1309–24. doi: 10.1007/s11764-023-01372-7 (PMC11324680; doi:10.1007/s11764-023-01372-7)
Supplement: Supplementary file 2 — List of references excluded during full-text review and reason for exclusion. [file 11764_2023_1372_MOESM2_ESM.pdf]

Online Resource 2 - List of references excluded during full-text review and reason for exclusion

Conference abstract (n = 7)

Vitelli et al., 2013. https://doi.org/10.1177/2047487314530052

Bucciarelli et al., 2017a. https://doi.org/10.1093/eurheartj/ehx501.P650

Bucciarelli et al., 2017b. https://esc365.escardio.org/presentation/162878

Bucciarelli et al., 2018. https://esc365.escardio.org/presentation/182734

Fong et al., 2015. http://dx.doi.org/10.1016/j.physio.2015.03.615

Lähteenmäki et al., 2012. https://doi.org/10.1002/pbc.24295

Maresca et al., 2013. https://doi.org/10.1177/2047487314530052

Article ineligible due to:

Outcome (n = 10)

Bell et al., 2021. https://doi.org/10.1007/s00520-021-06259-w

Bourke et al., 2018. https://doi.org/10.1038/s41598-018-26682-0

Dieli-Conwright et al., 2018. https://doi.org/10.1200/JCO.2017.75.7526

Fairey et al., 2005. https://doi.org/10.1016/j.bbi.2005.04.001

Gaskin et al., 2016. https://doi.org/10.1007/s11764-016-0543-6

Lee et al., 2019. https://doi.org/10.1001/jamaoncol.2019.0038

Lee et al., 2020. https://doi.org/10.1038/s41598-020-61927-x

Nikander et al., 2012. https://www.ismni.org/jmni/pdf/49/02NIKANDER.pdf

Uth et al., 2020. https://doi.org/10.1016/j.pcad.2020.08.002

Yen et al., 2019. https://doi.org/10.1007/s00520-019-04786-1

Outcome and Intervention (n = 2)

Anulika Aweto et al., 2015. https://www.ajol.info/index.php/nqjhm/article/view/177680

Lee et al., 2018. https://doi.org/10.1177/1534735418805149

Intervention (n = 1)

Giallauria et al., 2016. https://doi.org/10.1007/s11739-015-1259-8
