# Supplementary material for: Efficacy of exercise training for improving vascular dysfunction in people with cancer: a systematic review with meta-analyses
Source: J Cancer Surviv. 2023 Apr 20;18(4):1309–24. doi: 10.1007/s11764-023-01372-7 (PMC11324680; doi:10.1007/s11764-023-01372-7)
Supplement: Supplementary file 3 — Quality assessment tools risk-of-bias. [file 11764_2023_1372_MOESM3_ESM.pdf]

Online Resource 3 - Quality assessment tools risk-of-bias

**Cochrane Quality Assessment**							
	**Selection Bias**		**Performance Bias**	**Attrition Bias**	**Reporting Bias**	**Other bias**	
**Study**	Adequate allocation generation?	Allocation adequately concealed?	Adequate prevention of knowledge of allocation?	Incomplete outcome data adequately addressed?	Free of selective outcome reporting?	Free of other risks of bias?	**Quality Score**
Adams et al., 2017 [28]	Y	Y	Y	N	Y	N	66%
Ashton et al., 2021 [29]	Y	Y	Y	Y	Y	N	83%
Jones et al., 2013 [44]	Y	Y	Y	Y	Y	N	83%
Jones et al., 2014 [45]	Y	Y	Y	Y	Y	N	83%
Jones et al., 2020 [31]	Y	N	NR	Y	Y	Y	66%[a]
Lee at al., 2019 [30]	Y	Y	N	Y	N	Y	66%
Toohey et al., 2016 [43]	Y	NR	NR	Y	Y	N	50%[a]
Toohey et al., 2018 [32]	Y	NR	NR	N	Y	N	33%[a]
Wall et al., 2017 [33]	Y	Y	Y	Y	Y	Y	100%
Total Score	9/9	6/9	5/9	7/9	8/9	3/9	

[a] Quality rating calculated with missing information not reported in article
N = No; *NR* = Not Reported; *Y* = Yes

**Modified Newcastle-Ottawa Quality Appraisal**								
	**Selection Bias**	**Performance Bias**		**Detection Bias**		**Information Bias**		**Quality Score**
**Study**	Source population appropriate and representative?	Sample size adequate with sufficient power?	Identified and adjusted for any variables or confounders?	Appropriate statistical analysis methods?	Missing data? If so, handled accordingly?	Methodology of outcome measurement explicitly stated?	Objective assessment of outcome?	
Järvelä et al., 2013 [46]	2	1	3	3	2	2	3	76%
Long et al., 2018 [47]	2	1	0	3	3	3	3	71%

0 = Definitely no (high risk of bias); *1* = Mostly no; *2* = Mostly yes; *3* = Definitely yes (low risk of bias)
