# Supplementary material for: Efficacy of exercise training for improving vascular dysfunction in people with cancer: a systematic review with meta-analyses
Source: J Cancer Surviv. 2023 Apr 20;18(4):1309–24. doi: 10.1007/s11764-023-01372-7 (PMC11324680; doi:10.1007/s11764-023-01372-7)

Online Resource 4 - Meta-analyses funnel plots

a. Flow-mediated dilation funnel plot

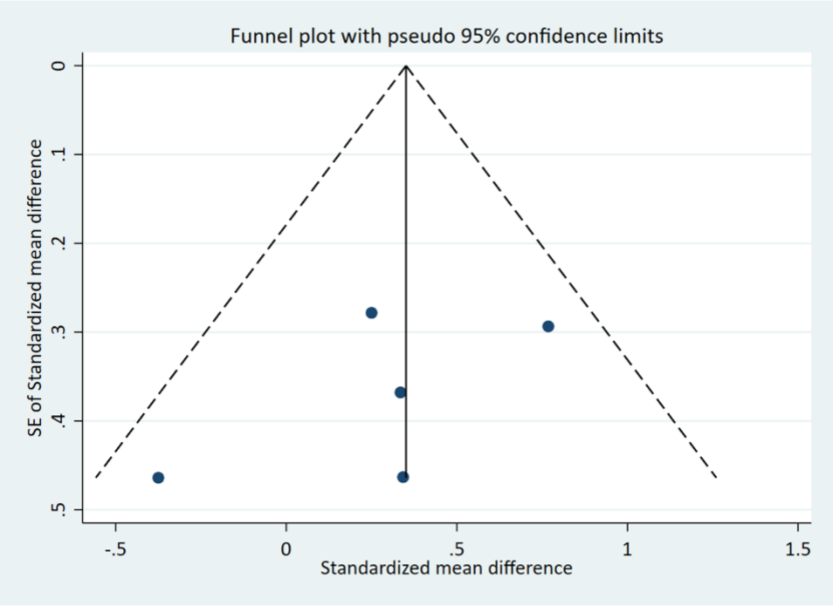

b. Pulse wave analysis funnel plot

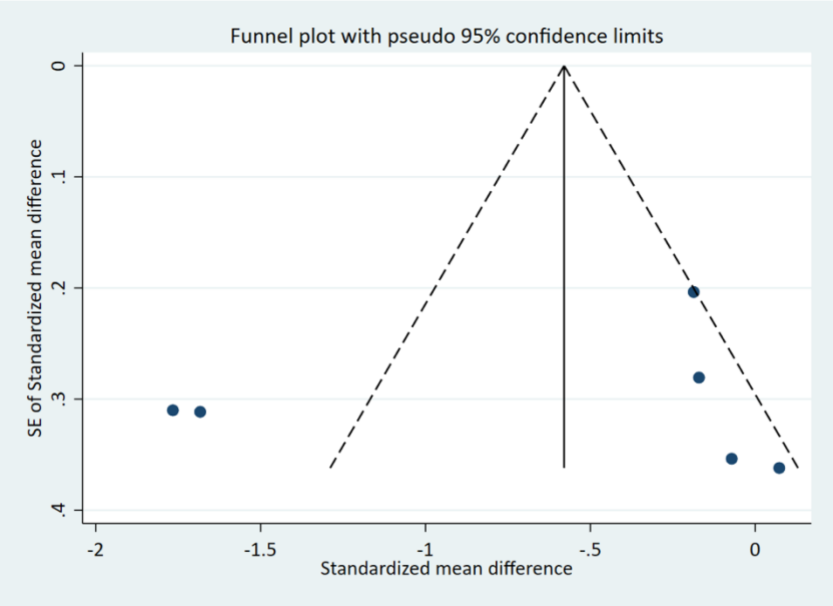

Supplement: Supplementary file 4 — Meta-analyses funnel plots. [file 11764_2023_1372_MOESM4_ESM.pdf]
