# Supplementary material for: Efficacy of exercise training for improving vascular dysfunction in people with cancer: a systematic review with meta-analyses
Source: J Cancer Surviv. 2023 Apr 20;18(4):1309–24. doi: 10.1007/s11764-023-01372-7 (PMC11324680; doi:10.1007/s11764-023-01372-7)
Supplement: Supplementary file 5 — Forest plot of pulse wave velocity in usual care vs. exercise intervention according to pulse wave velocity sub-group (central and peripheral), and overall combined. [file 11764_2023_1372_MOESM5_ESM.pdf]

Online Resource 5 - Forest plot of pulse wave velocity in usual care vs. exercise intervention according to pulse wave velocity sub-group (central and peripheral), and overall combined

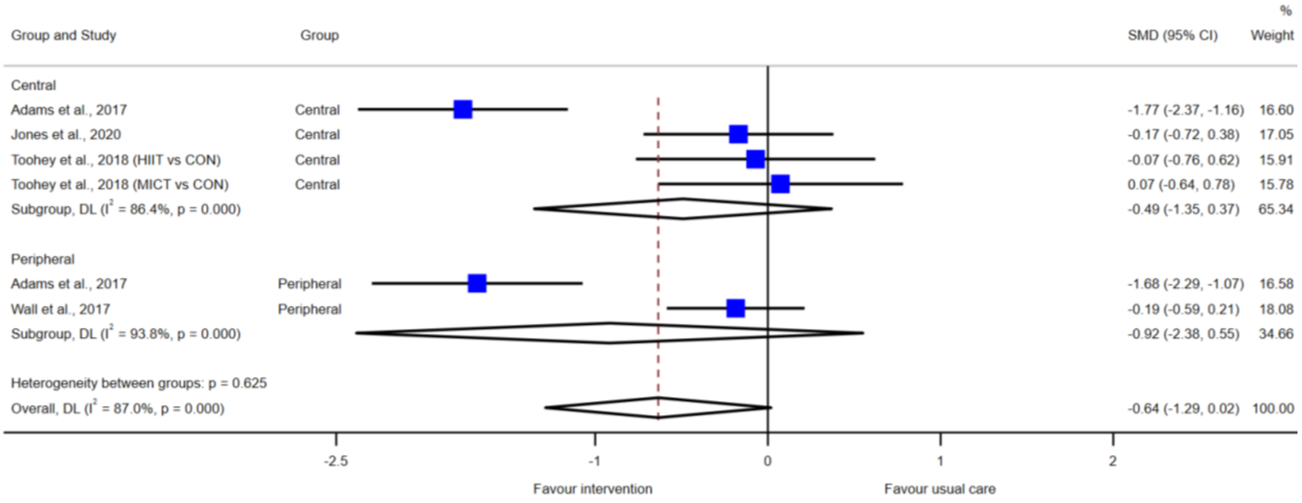

Data depicts the standardised mean differences and 95%CI for central, peripheral, and combined pulse wave velocity in individual studies and pooled estimates.

CI = confidence intervals; *CON* = control (usual care) group; *HIIT* = high-intensity interval training; *SMD* = standardised mean difference.
