# Supplementary material for: Efficacy of exercise training for improving vascular dysfunction in people with cancer: a systematic review with meta-analyses
Source: J Cancer Surviv. 2023 Apr 20;18(4):1309–24. doi: 10.1007/s11764-023-01372-7 (PMC11324680; doi:10.1007/s11764-023-01372-7)
Supplement: Supplementary file 6 — Individual domains and overall GRADE assessment. [file 11764_2023_1372_MOESM6_ESM.pdf]

Online Resource 6 - Individual domains and overall GRADE assessment

Certainty assessment							Summary of findings					
№ of studies	Study design	Risk of bias	Inconsistency	Indirectness	Imprecision	Other considerations	№ of patients		Effect		Certainty	Importance
							Exercise	Usual Care	Relative (95% CI)	Absolute (95% CI)		
**Flow-mediated Dilation (assessed with: Ultrasound)**												
5	randomised trials	not serious	not serious	not serious	serious [a]	none	89	82	-	SMD **0.34 SD higher** (0.01 higher to 0.67 higher)	⨁⨁⨁◯ Moderate	IMPORTANT
**Pulse Wave Velocity (assessed with: Ultrasound)**												
4	randomised trials	not serious	serious [b]	not serious	serious [c]	none	186	147	-	SMD **0.64 SD lower** (1.29 lower to 0.02 higher)	⨁⨁◯◯ Low	IMPORTANT

[a] Small total sample size
[b] Some concerns with CIs, and high heterogeneity

Source: GRADEpro Guideline Development Tool [Software]. McMaster University and Evidence Prime, 2022. Available from gradepro.org.
